# Salvianolic Acid B Attenuates Liver Fibrosis via Suppression of Glycolysis-Dependent m1 Macrophage Polarization

**DOI:** 10.3390/cimb47080598

**Published:** 2025-07-29

**Authors:** Hao Song, Ze-Wei Li, Wei Xu, Yang Tan, Ming Kuang, Gang Pei, Zhi-Qi Wang

**Affiliations:** 1 Pharmacy of College, Hunan University of Chinese Medicine, Changsha 410208, China; 20233772@stu.hnucm.edu.cn (H.S.); 1456992714lzw@stu.hnucm.edu.cn (Z.-W.L.); 15893347174@139.com (W.X.); 004746@hnucm.edu.cn (Y.T.); 004956@hnucm.edu.cn (M.K.); 2 Key Laboratory of Modern Research of TCM, Education Department of Hunan Province, Changsha 410208, China

**Keywords:** macrophages, Salvianolic acid B, glycolysis, liver injury

## Abstract

Liver fibrosis, a critical pathological feature of chronic liver injury, is closely associated with macrophage-mediated inflammatory responses and metabolic reprogramming. Blocking the fibrosis process will be beneficial to the treatment and recovery of the disease. Liver macrophages are a remarkably heterogeneous population of immune cells that play multiple functions in homeostasis and are central to liver fibrosis. Glycolysis-mediated macrophage metabolic reprogramming leads to an increase in the proportion of M1 macrophages and the release of pro-inflammatory cytokines. The present study aimed to investigate the therapeutic effect and mechanism of acid B (SAL B) against carbon tetrachloride (CCl_4_)-induced liver fibrosis. Here, we demonstrate that SAL B reduced the production of inflammatory factors in CCl_4_-induced liver fibrosis. Mechanistically, SAL B increased the expression of migration inhibitor 1 (MIG1) by inhibiting DNMT1-mediated methylation of the MIG1 promoter. Subsequently, MIG1 reduced the transcription of lactate dehydrogenase A (LDHA) and hexokinase 2 (HK2) which blocked glycolysis-mediated macrophage M1 polarization. In summary, our results suggested that SAL B is a promising intervention for ameliorating liver fibrosis.

## 1. Introduction

Chronic liver disease (CLD) is a major global health burden, causing approximately 2 million deaths worldwide each year [1]. The progression of CLD, irrespective of etiology, involves chronic parenchymal injury and persistent activation of inflammatory response as well as sustained activation of liver fibrogenesis and wound healing response [2]. Liver fibrosis results from chronic damage to the liver in conjunction with the accumulation of extracellular matrix (ECM) proteins, and advanced liver fibrosis results in cirrhosis, liver failure, and portal hypertension and often requires liver transplantation [3]. Current evidence highlights liver fibrosis as a maladaptive repair process sustained by chronic inflammatory cascades triggered by persistent inflammation [2]. This dysregulated inflammation transforms transient tissue repair into a self-amplifying cycle, marked by excessive extracellular matrix deposition and progressive architectural remodeling [4].

Hepatic macrophages are essential for maintaining tissue homeostasis and ensuring a rapid response to liver injury, and include Kupffer cells (KCs, liver-resident and self-maintaining macrophages) and monocyte-derived macrophages that accumulate in the injured liver [2]. These cells exhibit remarkable plasticity in response to injury signals and can adapt their polarization and can either promote restoration of tissue integrity following acute injury or, in case of chronic injury, contribute to CLD progression [5]. When stimulated by external pathogens, cytokines, and tumor metabolism, macrophages undergo polarization and metabolic reprogramming [6]. According to the difference in surface markers, gene expression patterns, and functions, macrophages are divided into M1 (classical) and M2 (alternative) phenotypes. M1 macrophages amplify inflammation via secretion of tumor necrosis factor-alpha (TNF-α) and interleukin-6 (IL-6), In contrast, M2 macrophages facilitate tissue repair by producing IL-10 and arginase-1 (ARG-1) [7].

The presentation of various phenotypes by macrophages is greatly influenced by metabolic reprogramming. Various stimuli induce intracellular alterations in metabolic patterns, known as metabolic reprogramming, such as glucose metabolism, lipid metabolism, and other metabolic pathways [8,9]. Macrophages acquire new phenotypes and functions through metabolic reprogramming to better adapt to different microenvironments [10]. In activated M1 macrophage cells, metabolism is shifted toward glycolysis and nitric oxide (NO) and citrulline production. This switch increases glucose uptake and lactate [11,12]. In M1 macrophages, the Krebs cycle intermediate succinate regulates hypoxia-inducible factor-1α (HIF-1α), driving sustained interleukin-1β (IL-1β) production [13]. M2 macrophages use oxidative phosphorylation for the more long-term functions involved in tissue repair and wound healing [14]. In M2 macrophages, glucose consumption is significantly lower as compared with M1 [12]. Therefore, changes in glycolipid metabolism in macrophages dictate M1/M2 polarization and regulate their immune functions.

Salvia miltiorrhiza, commonly known as Danshen in Chinese medicine, is the dried root of Salvia miltiorrhiza Bunge, which is a member of the Lamiaceae family and exhibits anti-inflammatory [15], antioxidant, and anti-fibrotic properties [16,17]. Its bioactive components are broadly classified into lipophilic diterpenoids (e.g., tanshinones) and hydrophilic phenolic acids, with SAL B being the most abundant and pharmacologically active water-soluble polyphenol [18]. However, the exact mechanism of whether SalB can regulate macrophage polarization by inhibiting glycolysis and thus alleviate hepatic fibrosis is still unknown. Using a CCl_4_-induced murine fibrosis model and lipopolysaccharide (LPS)-stimulated RAW264.7 macrophages, we demonstrate that SAL B upregulates MIG1 expression by reducing DNMT1-mediated methylation of its promoter. This epigenetic reprogramming inhibits the transcription of glycolytic genes, thereby blunting M1 polarization. Our study uncovers a novel metabolic-epigenetic axis through which SAL B exerts its anti-fibrotic effects.

## 2. Materials and Methods

### 2.1. Chemicals and Reagents

Salvianolic acid B (SAL B, CAS number 121521-90-2, purity ≥ 98%) was procured from Chengdu Push Biotechnology (Chengdu, China). 2-deoxy-D-glucose (2-DG) (HY-13966) and msc1094308 (HY-123872) was obtained from MedChemExpress. Lipopolysaccharides (LPS, Cat# L2630) were sourced from Sigma-Aldrich (Shanghai, China). The ATP assay kit (Cat# A095-1-1) and lactic acid assay kit (Cat# A019-2-1) were purchased from Nanjing Jiancheng Bioengineering Institute (Nanjing, China). The NAD^+^/NADH (Cat# S0175) assay kit was obtained from Beyotime Biotechnology (Beijing, China). AST/GOT activity assay kits (Cat# E-BC-K236-M), ALT/GPT activity assay kits (Cat# E-BC-K235-M), and hydroxyproline (Cat# E-BC-K062-S) were acquired from Elabscience (Wuhan, China). Antibodies against α-SMA (Cat# 14395-1-AP), MIG1 (Cat# 17673-1-AP), β-actin (Cat# 66009-1-Ig), LDHA (Cat# 19987-1-AP), HK2 (Cat# 22029-1-AP), ARG-1 (Cat# 16001-AP), and INOS (Cat# 18985-1-AP) were obtained from Proteintech (Wuhan, China). ELISA kits for IL-1β (Cat# EK201BHS), IL-6 (Cat# EK206HS), and TNF-α (Cat# EK282HS) were purchased from MULTISCIENCES (Hangzhou, China).

### 2.2. Cell Culture

RAW264.7 cells (CL-0190) and a specialized medium (CM-0190) were procured from Pri-cell (Wuhan, China). The cells were passaged using a pasteurized pipette, inoculated into 6-well plates at a density of 5 × 10^5^ cells per well, and incubated at 37 °C with 5% CO_2_. After 24 h of cultivation at 37 °C, the cells were induced with 1 μg/mL LPS for 24 h, treated with SAL B (1, 5, and 10 μM) for 24 h, and subsequently harvested.

### 2.3. Animal Experiments

In this study, male C57BL/6 mice, sourced from Slack Jingda Experimental Animal Company in Changsha, China, were utilized. At the time of experimentation, the mice were aged between 6 and 8 weeks and weighed approximately 20 to 22 g. The mice were maintained under controlled environmental conditions, which included a 12 h light/dark cycle and a temperature of 22 ± 2 °C. They were provided with ad libitum access to food and water. All experimental protocols were approved by the Institutional Ethical Committee on Animal Care and Experimentations at Hunan University of Chinese Medicine, Changsha (Approval Number: HNUCMZI-2407-15, Date: 15 July 2024), adhering to the guidelines outlined in the “Guide for the Care and Use of Laboratory Animals” published by the US National Institutes of Health, National Research Council (2011, 8th ed., National Academies Press, ISBN 978-0-309-15400-0). Following a one-week acclimatization period, the mice were randomly assigned to six groups, each consisting of 10 mice: the control group, the CCl_4_ group, and three treatment groups receiving low (10 mg/kg, i.g.), medium (20 mg/kg, i.g.), and high (40 mg/kg, i.g.) doses of SAL B. The mice were administered their respective treatments or an equivalent volume of saline intragastrically each day between 9:00 and 10:00 a.m. for a duration of 42 days. Except for the control group, all groups received subcutaneous injections of 10% CCl_4_ (dissolved in corn oil) at a dosage of 0.1 mL/10 g body weight, three times per week for 6 weeks. The control group received isopycnic corn oil injections. Following a 1-week acclimatization period, the mice were randomly assigned to six groups, each consisting of 10 mice: the control group; the CCl_4_ group; and three treatment groups receiving low (10 mg/kg, i.g.), medium (20 mg/kg, i.g.), and high (40 mg/kg, i.g.) doses of SAL B. The mice were administered their respective treatments or an equivalent volume of saline intragastrically each day between 9:00 and 10:00 a.m. for a duration of 42 days. Except for the control group, all groups received subcutaneous injections of 10% CCl_4_ (dissolved in corn oil) at a dosage of 0.1 mL/10 g body weight, three times per week for 6 weeks. The control group received isopycnic corn oil injections.

### 2.4. Blood Biochemical Test

To collect blood samples from mice, the animals were initially restrained by gently pinching the skin behind their ears, followed by trimming the whiskers on the designated blood collection side. Using blood sampling from the eye socket, allowing the blood to drip directly into an EP tube. The collected samples were incubated at room temperature for 4 h. Blood samples were centrifuged for 10 min at 3500 rpm to separate the serum. The biomarkers of serum alanine aminotransferase (ALT), aspartate transaminase (AST), and hydroxyproline (Hyp) were measured using kits from Nanjing Jiancheng Bioengineering Institute (Nanjing, China).

### 2.5. Histopathological Analysis

After fixation, liver tissues were embedded in paraffin and sectioned into 5 μm thick slices. The liver paraffin sections were stained with H&E. Additionally, Masson and Sirius red staining was performed to assess collagen and muscle fiber alterations. Each microscopic field was examined under a light microscope.

### 2.6. Quantitative Real-Time PCR Analysis (qRT-PCR)

After treatment with or without LPS (1 μg/mL) and SAL B (1, 5, 10 μM) at 37 °C for 24 h, total RNA was isolated and quantified using the Simgen blood/cultured cell DNA kit. Total RNA was reverse-transcribed to cDNA using the Reverse Transcription Kit. Relative gene expressions were detected using the ABI7500 platform (Thermo Fisher Scientific, Waltham, MA, USA) with SYBR Green Master Mix. The RT-PCR primer sequences are listed in Table 1.

### 2.7. Western Blot Analysis

Cells or liver tissues were collected and lysed with RIPA buffer for 30 min, followed by centrifugation at 13,000 rpm at 4 °C for 10 min. After protein quantification with the BCA kit, the samples were mixed with a loading buffer. Proteins MIG1 (1:1000, 17673-1-AP), β-actin (1:10,000, 66009-1-Ig), LDHA (1:10,000, 19987-1-AP), HK2 (1:20,000, 22029-1-AP), ARG-1 (1:5000, 16001-AP), and INOS (1:4000, 18985-1-AP) were separated by SDS-PAGE using gels ranging from 8% to 12%. Protein expressions were detected by chemiluminescence.

### 2.8. Molecular Docking

The crystal structure of DNMT1 (PDB code: 3AV4) was selected as the docking receptor as previously reported [19]. After adding missing atoms, deleting water molecules, and performing energy minimization with Autodock Vina 1.1.2, the 3D structure of SAL B was prepared for docking with the DNMT1 structure under default parameters. The interaction between SAL B and DNMT1 was visualized using PyMOL 2.5.0 (Schrödinger, Portland, OR, USA).

### 2.9. Detection of DNA Methylation Levels

After treatment with or without LPS (1 μg/mL) and SAL B (1, 5, 10 μM) at 37 °C for 24 h, total DNA was isolated and quantified using the Simgen blood/cultured cell DNA kit. Relative DNA modifications were detected using the DNA Methylation Extreme Modification Kit with SYBR Green Master Mix. The RT-PCR primer sequences are listed in Table 2.

### 2.10. Statistical Analysis

Results are presented as the mean ± standard error of the mean (SEM). Data analysis was conducted utilizing a one-way analysis of variance (ANOVA) with Prism 9.5 software (GraphPad Software, La Jolla, CA, USA). Protein abundance in Western blot analyses was quantified using ImageJ software 9 (specifically ImageJ 1.53t). All bar graphs were generated utilizing Prism version 9.5. Statistical significance was assessed with a threshold of *p* < 0.05. A *p*-value less than 0.05 is considered statistically significant, whereas a *p*-value less than 0.01 is considered highly significant. Levels of significance are indicated as follows: * for *p* < 0.05 and ** for *p* < 0.01.

## 3. Results

### 3.1. SAL B Ameliorated Liver Fibrosis and Liver Injury in CCl_4_-Treated Mice

**Figure 1:**
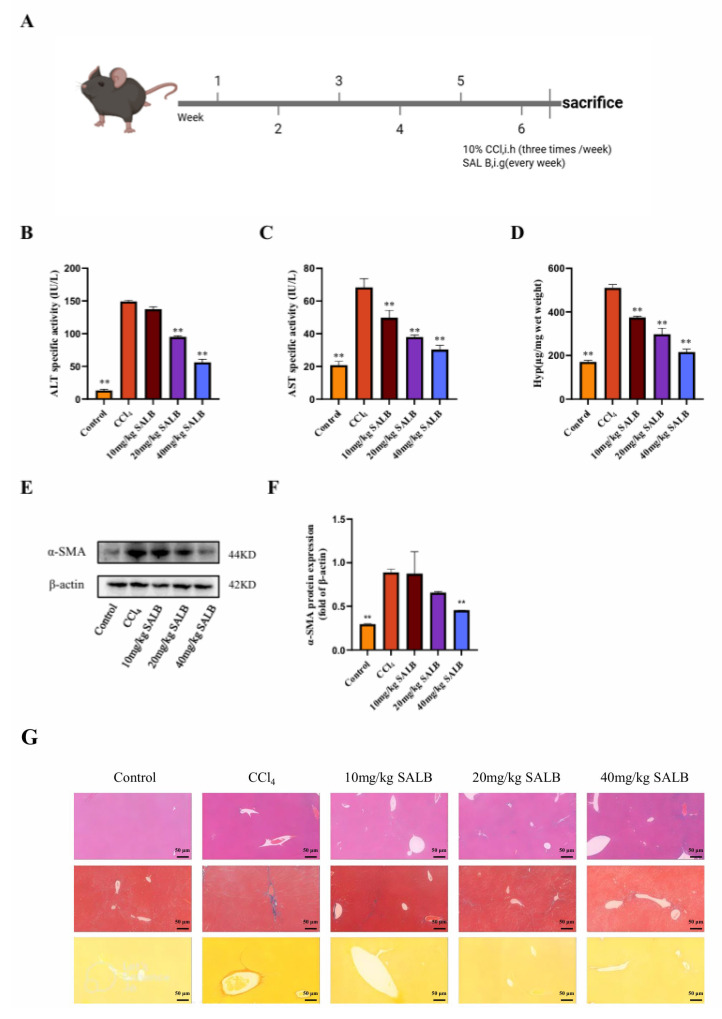
SAL B ameliorated liver fibrosis and liver injury in CCl_4_ mice. (**A**) Schematic representation of the CCl_4_-induced liver fibrosis model and treatment timeline. (**B**–**D**) The levels of ALT, AST, and Hyp were measured by ELISA in serum and liver tissues in different groups with CCl_4_ or Corn Oil and SAL B therapy. (**E**,**F**) Western blot analysis to detect the expression of α-SMA and β-actin in liver tissues in different groups with CCl_4_ or Corn Oil and SAL B therapy. (**G**) Representative images of HE, Masson, and Sirius red staining in liver tissues in different groups were performed. Data are presented as means ± SEM. (*n* = 3). ** *p* < 0.01. Data are representative of three independent experiments with similar results.

To delineate the anti-fibrotic mechanism of SAL B, we employed the established CCl_4_-induced murine liver fibrosis model. Mice received subcutaneous CCl_4_ injections every 3 days. Treatment groups concurrently received daily intraperitoneal SAL B (10, 20, or 40 mg/kg), while control and model groups received the saline vehicle. After 6 weeks (Figure 1A), fibrosis induction was confirmed.

SAL B’s therapeutic efficacy was evaluated through multiple parameters. Serum ALT, AST, and Hyp levels, markers of hepatocyte injury and fibrogenesis, were significantly elevated in model mice. SAL B treatment dose-dependently attenuated these elevations (Figure 1B–D). Consistently, liver α-SMA protein expression (a fibrosis indicator) increased markedly in model mice, an effect reversed by SAL B (Figure 1E,F). Histopathological analysis via H&E staining revealed substantial immune cell infiltration and hepatocyte necrosis in model livers, alongside pronounced collagen deposition and fibroblast proliferation. SAL B administration mitigated all pathological features (Figure 1G). Collectively, these data demonstrate SAL B’s potent protective effects against CCl_4_-induced hepatic injury and fibrogenesis.

### 3.2. SAL B Mitigated Polarization and Glycolysis Levels of Liver Macrophages in the CCl_4_ Mice Model

**Figure 2:**
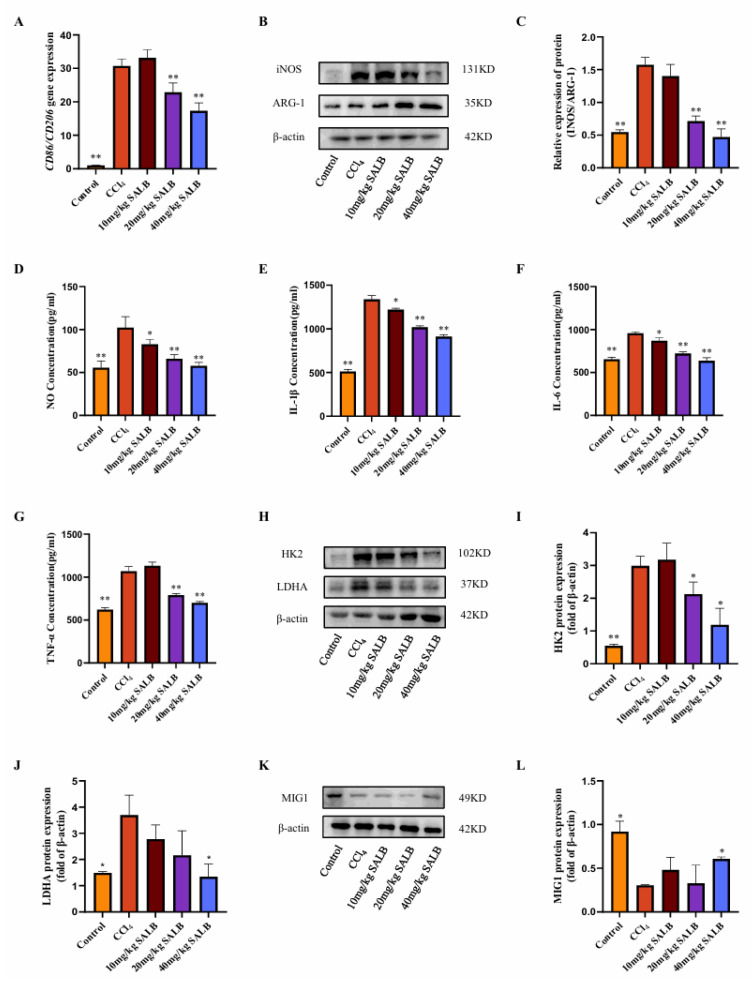
SAL B mitigated polarization and glycolysis levels of liver macrophages in the CCl_4_ mice model. (**A**) qRT-PCR analysis of *Cd86* and *Cd206* mRNA in liver tissues in different groups with CCl_4_ or Corn Oil and SAL B therapy. (**B**,**C**) Western blot analysis to detect the expression of INOS and ARG-1 in liver tissues in different groups with CCl_4_ or Corn Oil and SAL B therapy. (**D**) NO secretions were determined by Griess assay, respectively, in liver tissues in different groups with CCl_4_ or Corn Oil and SAL B therapy. (**E**–**G**) IL-1β, IL-6, and TNF-α secretions were determined by ELISA, respectively, in liver tissues in different groups with CCl_4_ or Corn Oil and SAL B therapy. (**H**–**J**) Western blot analysis to detect the expression of HK2 and LDHA in liver tissues in different groups with CCl_4_ or Corn Oil and SAL B therapy. (**K**,**L**) Western blot analysis to detect the expression of MIG1 in liver tissues in different groups with CCl_4_ or Corn Oil and SAL B therapy. Data are presented as means ± SEM. (*n* = 3). * *p* < 0.05, ** *p* < 0.01. Data are representative of three independent experiments with similar results.

In fibrotic livers, the infiltration of inflammatory cells is a critical factor in the advancement of fibrosis. The polarization state of macrophages in liver fibrosis significantly influences fibrosis progression. M1-type macrophages rapidly generate energy through aerobic glycolysis and facilitate the release of inflammatory factors. Consequently, we investigated alterations in macrophage glycolysis and M1 polarization using the CCl_4_ model. We assessed the gene expression of M1- and M2-type macrophage markers, *Cd86* and *Cd206*, respectively, in the livers of mice across different groups (Figure 2A). The findings revealed a significant increase in the ratio of M1-type to M2-type macrophage markers in the livers of mice in the model group. SAL B markedly inhibited this alteration, and Western blot analysis of the M1-type marker inducible nitric oxide synthase (INOS) and the M2-type marker ARG-1 corroborated these results (Figure 2B,C). Furthermore, enzyme-linked immunosorbent assay (ELISA) was employed to measure the levels of NO released by M1-type macrophages and the expression levels of inflammatory cytokines IL-1β, IL-6, and TNF-α in the liver.

The administration of SAL B significantly decreased the release levels of NO, IL-1β, IL-6, and TNF-α in a dose-dependent manner (Figure 2D–G). This suggests that SAL B reduces the prevalence of inflammatory phenotypes in macrophages, consequently diminishing the secretion of inflammatory mediators and ameliorating liver inflammation. Furthermore, SAL B treatment markedly inhibited the protein expression of key glycolytic enzymes, HK2 and LDHA, in the livers of mice (Figure 2H–J).

Transcriptional regulation of glycolysis is fundamentally governed by the MYC oncogene, which directly activates key glycolytic enzymes, including HK2 and LDHA [20,21]. Beyond MYC, recent research has identified MIG1 as a critical coordinator of nuclear-mitochondrial communication, regulating glycolysis specifically in colorectal cancer [22]. In Saccharomyces cerevisiae, the zinc finger protein MIG1 functions as a well-established glucose metabolic regulator, primarily mediating glucose repression [23]. Our Western blot analysis revealed that MIG1 protein expression levels were diminished in the liver of the model group, but were significantly restored following SAL B treatment (Figure 2K,L). In conclusion, our experimental findings suggest that SAL B treatment modulates the polarization and glycolytic activity of liver macrophages in mice treated with CCl_4_, thereby inhibiting the production of inflammatory factors.

### 3.3. SAL B Reduced the Inflammatory Responses and Polarization of Macrophages Stimulated by LPS

**Figure 3:**
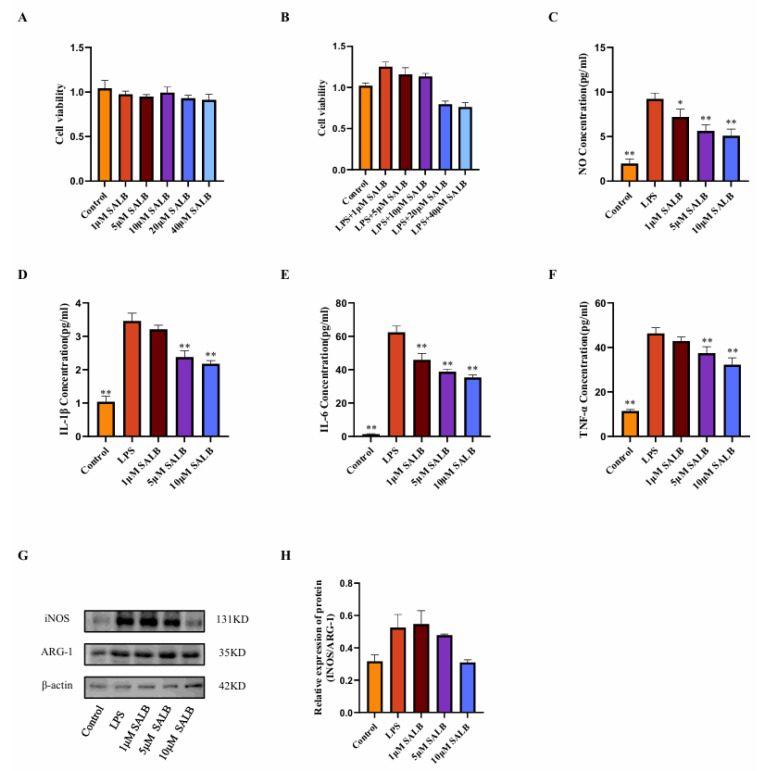
SAL B reduced the inflammatory responses and polarization of macrophages stimulated by LPS. (**A**) Cell viability measured by CCK8 assay after SAL B treatment with RAW 264.7 at various concentrations (0, 1, 5, 10, 20, 40 µM). (**B**) Cell viability measured by CCK8 assay after LPS and SAL B treatment with RAW 264.7 at various concentrations (0, 1, 5, 10, 20, 40 µM). (**C**) NO secretions were determined by Griess assay, respectively, in cell culture medium with RAW 264.7 LPS or non-treated (NT) and SAL B therapy. (**D**–**F**) IL-1β, IL-6, and TNF-α secretions were determined by ELISA, respectively, in cell culture medium with RAW 264.7 LPS or NT and SAL B therapy. (**G**,**H**) Western blot analysis to detect the expression of INOS and ARG-1 in RAW 264.7 LPS or NT and SAL B therapy. Data are presented as means ± SEM. (*n* = 3). * *p* < 0.05, ** *p* < 0.01. Data are representative of three independent experiments with similar results.

To assess whether SAL B can inhibit macrophage polarization and glycolysis, we established an in vitro model of M1-type macrophages by stimulating RAW264.7 cells with 1 μg/mL LPS. First, the appropriate drug dose was determined using the CCK8 assay. Based on the literature review, a drug dose gradient was set, and it was found that treatment with 1, 5, 10, 20, and 40 μM of SAL B for 24 h did not affect the cell viability of RAW264.7 cells. When cells were co-treated with 1 μg/mL LPS and different concentrations of SAL B for 24 h, it was found that 20 and 40 μM SAL B reduced cell viability (Figure 3A,B). Based on the above experimental results, SAL B was administered at doses of 1, 5, and 10 μM. Following LPS stimulation, the release of NO by macrophages significantly increased, indicating macrophage polarization toward the M1 type. SAL B at doses of 1, 5, and 10 μM dose-dependently reduced the expression levels of NO, a marker of M1-type macrophage polarization (Figure 3C).

The concentrations of inflammatory mediators IL-1β, IL-6, and TNF-α present in the cell supernatant were quantified using ELISA. A dose-dependent reduction in the expression levels of these inflammatory mediators was observed (Figure 3D–F). The impact of SAL B on macrophage polarization was further corroborated through Western blot analysis. The expression levels of macrophage polarization markers, INOS, and ARG-1 were assessed. SAL B administration resulted in the inhibition of INOS expression, whereas ARG-1 expression remained unaffected. Consequently, the INOS/ARG-1 ratio exhibited a general decreasing trend (Figure 3G,H), suggesting that SAL B selectively diminishes the proportion of M1-type macrophages without altering M2-type macrophages. In conclusion, within an in vitro LPS-stimulated M1 pro-inflammatory macrophage model, SAL B attenuates M1-type polarization and reduces the secretion of inflammatory mediators by macrophages, thereby mitigating the inflammatory phenotype.

### 3.4. SAL B Inhibited M1 Macrophage Polarization by Negatively Regulating Glycolysis Levels

Previous research established that SAL B suppresses macrophage polarization toward the pro-inflammatory M1 phenotype. Macrophages switch their metabolism from oxidative phosphorylation to glycolysis when exposed to an external stimulus in a process known as inflammatory activation. To delineate the mechanistic basis of SAL B’s action, we quantified key glycolysis-related metabolites and key enzymes in LPS-induced M1 macrophages at 24 h post-stimulation.

**Figure 4:**
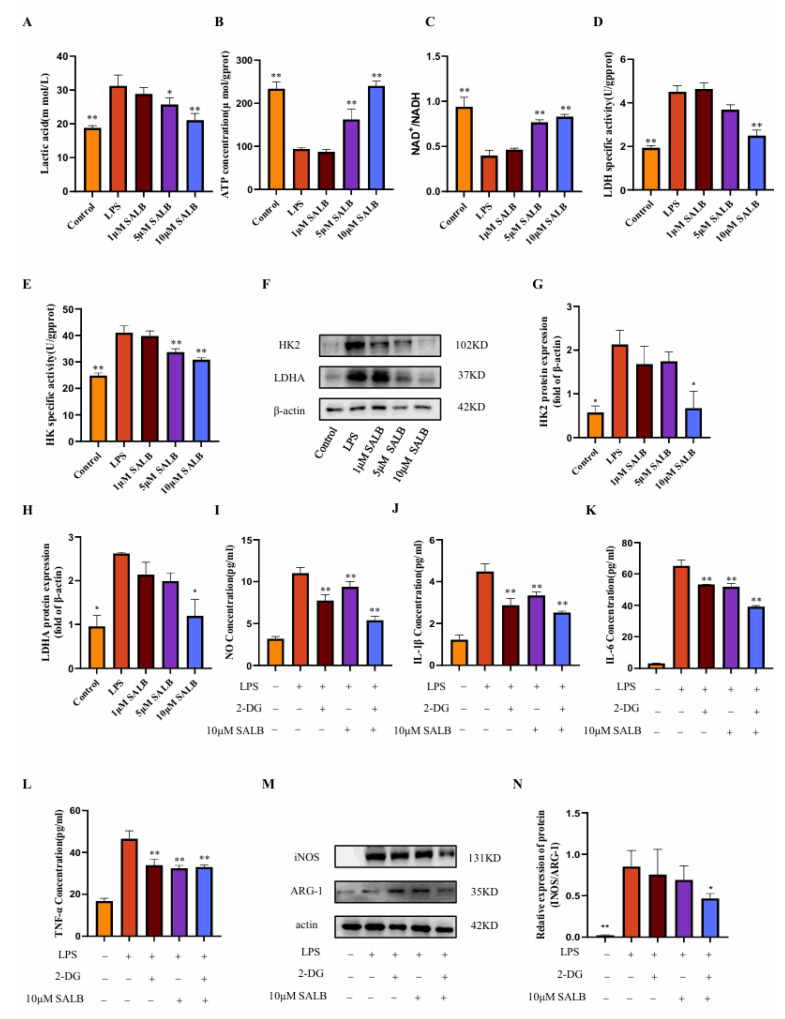
SAL B inhibited M1 macrophage polarization by negatively regulating glycolysis levels. (**A**) Lactate secretions were determined by Colorimetric method assay, respectively, in the cell supernatant after treatment with LPS or NT and SAL B therapy for 24 h. (**B**,**C**) ATP and NAD^+^/NADH secretions were determined by Colorimetric method and WST-8 assay, respectively, in the cell lysate after treatment with LPS or NT and SAL B therapy for 24 h. (**D**,**E**) The enzyme activity levels of HK and LDHA were measured in the cell lysate after treatment with LPS or NT and SAL B therapy for 24 h. (**F**–**H**) Western blot analysis to detect the expression of HK and LDHA in the cell lysate after treatment with LPS or NT and SAL B therapy for 24 h. (**I**) NO secretions were determined by Griess assay, respectively, in the cell supernatant after treatment with LPS or NT and SAL B therapy for 24 h. (**J**–**L**) IL-1β, IL-6, and TNF-α secretions were determined by ELISA, respectively, in the cell supernatant after treatment with LPS or NT and SAL B therapy for 24 h. (**M**,**N**) Western blot analysis to detect the expression of INOS and ARG-1 in RAW 264.7 LPS or NT and SAL B therapy. Data are presented as means ± SEM. (*n* = 3). * *p* < 0.05, ** *p* < 0.01. Data are representative of three independent experiments with similar results.

Initially, we measured lactate concentration, the terminal metabolite of aerobic glycolysis (Figure 4A). LPS stimulation markedly elevated lactate levels, whereas SAL B treatment induced a dose-dependent reduction in lactate accumulation. This finding directly indicates that SAL B effectively attenuates glycolytic flux. Complementing these metabolic observations, we assessed alterations in cellular energy status. LPS treatment significantly decreased both ATP levels and the NAD^+^/NADH ratio (Figure 4B,C). Notably, SAL B administration counteracted these perturbations, dose-dependently elevating ATP concentrations and restoring the NAD^+^/NADH ratio. These data collectively demonstrate that SAL B rescues the LPS-impaired cellular energy production capacity.

To identify potential molecular targets, we evaluated the activity of pivotal glycolytic enzymes. SAL B treatment significantly suppressed the enzymatic activity of HK2, which catalyzes the first committed glycolytic step, and LDHA, responsible for terminal lactate generation (Figure 4D,E). Further mechanistic investigation revealed that SAL B also dose-dependently diminished the protein expression levels of both HK2 and LDHA (Figure 4F–H).

To establish a causal linkage between glycolytic inhibition and anti-inflammatory efficacy, we employed 2-deoxy-D-glucose (2-DG), a specific glycolysis inhibitor [24]. Consistent with SAL B’s effects, both 2-DG and SAL B (10 μM) significantly attenuated the release of the M1 polarization marker NO (Figure 4I) and key pro-inflammatory cytokines (IL-1β, IL-6, TNF-α) (Figure 4J–L). Of significant mechanistic relevance, combined 2-DG and SAL B treatment exerted a more potent inhibitory effect on macrophage polarization and cytokine release than either agent alone. This functional synergy was further corroborated by Western blot analysis demonstrating synergistic suppression of M1 markers (Figure 4M,N). Collectively, these findings demonstrate that SAL B ameliorates LPS-induced M1 macrophage polarization and inflammatory mediator secretion primarily through targeted inhibition of glycolytic metabolism.

### 3.5. SAL B Reduced Glycolysis by Upregulating MIG1

**Figure 5:**
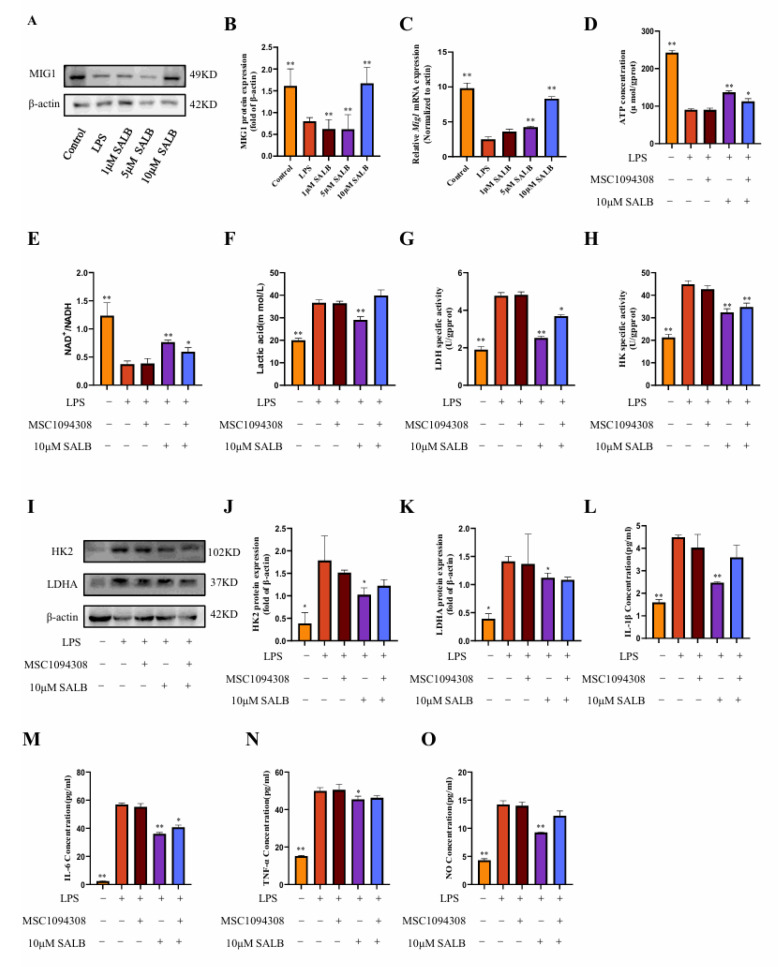
SAL B reduced glycolysis by upregulating MIG1. (**A**,**B**) Western blot analysis to detect the expression of MIG1 in the cell lysate after treatment with LPS or NT and SAL B therapy for 24 h. (**C**) qRT-PCR analysis of *Mig1* mRNA in the cell lysate after treatment with LPS or NT and SAL B therapy for 24 h. (**D**,**E**) ATP and NAD^+^/NADH secretions were determined by Colorimetric method and WST-8 assay, respectively, in the cell lysate after treatment with LPS or NT, MSC1094308, and SAL B therapy for 24 h. (**F**) Lactate secretions were determined by Colorimetric method assay, respectively, in the cell supernatant after treatment with LPS or NT, MSC1094308, and SAL B therapy for 24 h. (**G**,**H**) The enzyme activity levels of HK and LDHA were measured in the cell lysate after treatment with LPS or NT and SAL B therapy for 24 h. (**I**–**K**) Western blot analysis to detect the expression of HK and LDHA in the cell lysate after treatment with LPS or NT, MSC1094308, and SAL B therapy for 24 h. (**L**–**N**) IL-1β, IL-6, and TNF-α secretions were determined by ELISA, respectively, in the cell supernatant after treatment with LPS or NT, MSC1094308, and SAL B therapy for 24 h. (**O**) NO secretions were determined by Griess assay, respectively, in the cell supernatant after treatment with LPS or NT, MSC1094308, and SAL B therapy for 24 h. Data are presented as means ± SEM. (*n* = 3). * *p* < 0.05, ** *p* < 0.01. Data are representative of three independent experiments with similar results.

To assess the regulatory mechanism of SAL B on macrophage glycolysis, we found that the glycolysis transcription factor MIG1 showed a dose-dependent response to SAL B administration. To define SAL B’s regulatory role on MIG1 in vitro, we quantified MIG1 expression using qRT-PCR and Western blot analysis. These assays revealed that SAL B treatment significantly increased MIG1 expression at both mRNA and protein levels (Figure 5A–C).

To investigate the functional contribution of MIG1 to macrophage immunometabolism, we utilized the effective MIG1 inhibitor MSC1094308 [25]. Co-treatment with MSC1094308 and SAL B significantly abrogated the effects of SAL B on key glycolytic parameters: the SAL B-induced increases in ATP concentration and NAD^+^/NADH ratio were reversed, and the reduction in lactate levels was attenuated (Figure 5D–F). Furthermore, MSC1094308 antagonized the inhibitory effects of SAL B on the pivotal glycolytic enzymes HK2 and LDHA. Specifically, the inhibitor blocked SAL B-mediated suppression of both HK2 and LDHA enzymatic activity (Figure 5G–I) and diminished the reduction in HK2 and LDHA protein expression levels induced by SAL B (Figure 5J,K).

Subsequently, we assessed the impact of MIG1 inhibition on inflammatory outputs. Notably, treatment with MSC1094308 partially eliminated the suppressive effects of SAL B on the secretion of the M1 polarization marker NO and the pro-inflammatory cytokine IL-1β (Figure 5L,O). In contrast, MSC1094308 did not antagonize SAL B’s inhibitory effects on IL-6 and TNF-α release (Figure 5M,N). Collectively, these findings demonstrate that MIG1 is a target through which SAL B exerts its inhibitory effects on macrophage glycolytic metabolism and regulates inflammatory mediators.

### 3.6. SAL B Reduced the Methylation Level of the MIG1 Promoter Region by Decreasing DNMT1 Expression

**Figure 6:**
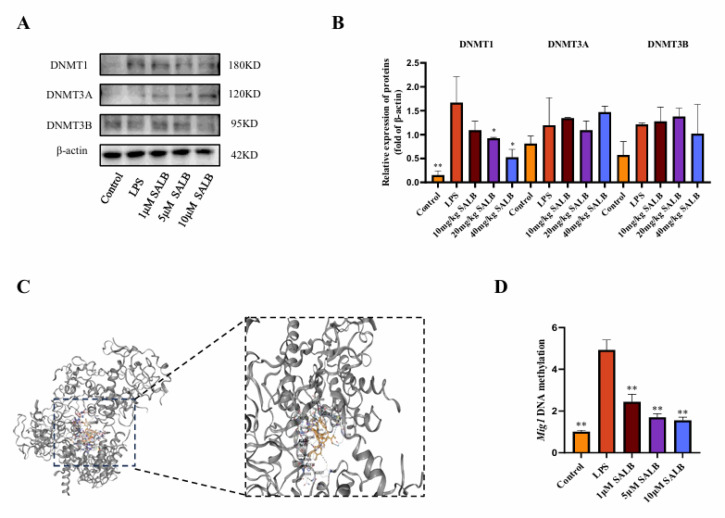
SAL B reduced the methylation level of the MIG1 promoter region by decreasing DNMT1 expression. (**A**,**B**) Western blot analysis to detect the expression of DNMT3A, DNMT3B, and DNMT1 in the cell lysate after treatment with LPS or NT and SAL B therapy for 24 h. (**C**) The interaction mode between DNMT1 and SAL B was characterized to understand their binding mechanism. (**D**) qRT-PCR analysis of MIG1 methylation level after treatment with LPS or NT and SAL B therapy for 24 h. Data are presented as means ± SEM. (*n* = 3). * *p* < 0.05, ** *p* < 0.01. Data are representative of three independent experiments with similar results.

The MIG gene family has a close regulatory relationship with DNA epigenetic modification inheritance (especially DNA methylation), which makes MIG play a key role in metabolism-related diseases. To investigate whether SAL B promotes the expression of MIG1 through DNA methylation, we assessed the expression of DNA methyltransferases by protein blotting analysis (Figure 6A,B). Among the three major DNA methyltransferases in mammals—DNMT1, DNMT3A, and DNMT3B [26], we found that under LPS stimulation, the protein expression of DNMT1, DNMT3A, and DNMT3B increased to varying degrees. Under the influence of SAL B, the protein expression of DNMT1 was significantly reduced, while there was no significant effect on the protein expression of DNMT3A and DNMT3B. Through molecular docking experiments, SAL B interacts with multiple amino acid residues of the protein DNMT1 via hydrogen bonds (Figure 6C). Docking score: −9.8. To further investigate the effect of SAL B on MIG1 methylation expression, we used sodium bisulfite conversion detection to assess MIG1 DNA methylation levels. The results demonstrated that SAL B treatment led to a reduction in MIG1 DNA methylation levels (Figure 6D). Together, these results indicate that SAL B reduces the DNA methylation level of MIG1 by inhibiting the expression of the methyltransferase DNMT1, thereby enhancing the expression level of MIG1.

## 4. Discussion

The CCl_4_-induced model of hepatic fibrosis in experimental animals remains a cornerstone methodology for elucidating the pathogenic mechanisms underlying human liver fibrosis, effectively recapitulating key disease progression stages [27]. Central to this process is hepatocellular injury, which instigates a pronounced influx of inflammatory cells into the hepatic parenchyma and the concomitant release of a spectrum of pro-inflammatory cytokines [28]. These molecular and cellular events constitute fundamental drivers initiating the fibrogenic cascade. Within the diverse immune cell infiltrate, macrophages, particularly those polarized toward the classically activated (M1) phenotype, assume a pivotal regulatory role in orchestrating the hepatic inflammatory milieu. It is well-established that inflammatory processes serve as the primary instigators of fibrogenesis, with the dynamic polarization of macrophages representing a critical determinant within the evolving inflammatory microenvironment. Exhibiting remarkable functional plasticity, macrophages possess the capacity to undergo phenotype switching, thereby modulating their effector functions in direct response to local environmental cues and signaling molecules [29]. This plasticity renders them both key drivers of pathology and potential therapeutic targets. Substantial clinical investigations and robust experimental data converge to underscore the central role of M1 macrophages as principal orchestrators and amplifiers of the pro-inflammatory response during the initiation and progression of liver fibrosis [29]. Their persistent activation creates a self-perpetuating cycle of inflammation and tissue damage that fuels fibrogenesis.

Consequently, therapeutic strategies targeting the pro-inflammatory M1 macrophage phenotype represent a primary focus for mitigating liver injury and fibrosis, based on the premise that reducing M1 polarization impedes disease progression. Modulating macrophage polarization, particularly shifting the balance away from the M1 state, is thus a rational and actively pursued approach. Metabolic changes play a crucial role in determining the activation, differentiation, and functions of immune cells, with macrophages being no exception [30]. M2 macrophages mainly rely on OXPHOS, while M1 macrophages shift to glycolysis, similar to the Warburg effect in tumors [31]. Although less efficient than OXPHOS, glycolysis meets the high energy needs of activated M1 macrophages due to its fast ATP production.

Given glycolysis’s role in inflammatory diseases, strategies have been developed to adjust glycolysis and alter macrophage behavior, such as in OA-related synovitis [32]. Several transcription factors are involved in the intricate and dynamic processes of immunometabolism. Our research has identified MIG1 as an upstream regulator that plays a critical role in the downregulation of glycolysis. In our in vitro experiments, the pharmacological activation of MIG1 effectively suppressed the abnormal elevation of glycolytic enzymes, excessive M1 polarization, and the increased expression of inflammatory genes. SAL B, a bioactive polyphenol derived from Salvia miltiorrhiza, has emerged as a compound of significant interest due to its anti-inflammatory and hepatoprotective properties [33]. SAL B suppresses hepatic stellate cells activation and liver fibrosis via regulation of miR-6499-3p/LncRNA-ROR-mediated nuclear factor kappa-B signaling pathway [34]. Here, we demonstrated a novel mechanism underlying SAL B’s anti-fibrotic action: SAL B increased the expression of MIG1 by inhibiting DNMT1-mediated methylation of the MIG1 promoter. Subsequently, MIG1 reduced the transcription of LDHA and HK2, which blocked glycolysis-mediated macrophage M1 polarization to mitigate liver fibrosis-associated inflammation.

Metabolic reprogramming critically governs immune cell ontogeny and function, highlighting its therapeutic potential for immune disorders. Macrophages exhibit metabolic plasticity, shifting from oxidative phosphorylation to glycolysis upon stimulation, with M1 polarization characterized by robust glycolytic activation. The transcription factor MIG1 promotes gluconeogenesis while repressing glycolysis [17]. Our study, utilizing both cellular models and preclinical liver fibrosis studies, reveals that SAL B enhances MIG1 expression via demethylation-mediated epigenetic regulation. Consequently, SAL B suppresses key glycolytic enzymes and associated genes, attenuates glycolytic flux, and mitigates inflammatory responses (Figure 2, Figure 3, Figure 4, Figure 5 and Figure 6). These findings elucidate a novel mechanism for the hepatoprotective effects of SAL B through the modulation of M1 macrophage polarization in liver fibrosis.

## 5. Conclusions

**Figure 7:**
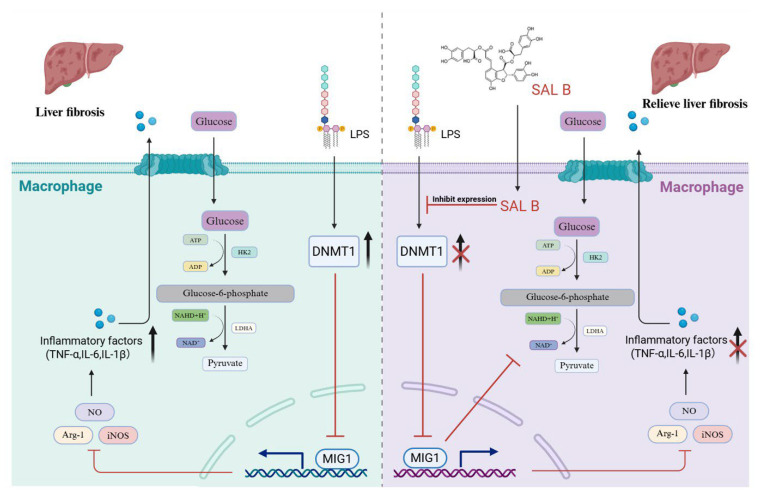
Schematic diagram. The present study demonstrated that SAL B significantly ameliorated liver fibrosis-associated inflammation and reduced the expression of inflammatory factors. This protective effect was achieved through inhibition of macrophage M1 polarization and glycolysis. SAL B upregulated the transcription factor MIG1, which inhibited the expression of key glycolytic proteins, thereby reducing macrophage M1 polarization and the release of inflammatory factors.

In summary, this study demonstrated that SAL B significantly improved liver fibrosis-related inflammation and reduced the expression of inflammatory factors. In this study, we found that SAL B increased the expression of MIG1 by inhibiting DNMT1-mediated MIG1 promoter methylation. The transcription factor MIG1 alleviated liver injury and fibrosis by blocking glycolysis-mediated macrophage M1 polarization by downregulating the transcription of LDHA and HK2 (Figure 7).

## Figures and Tables

**Table 1:** Primer sequences for real-time RT-PCR.

Gene	Forward Primer (5′–3′)	Reverse Primer (5′–3′)
*Cd86*	TGTTTCCGTGGAGACGCAAG	TTGAGCCTTTGTAAATGGGCA
*Cd206*	GAGGGAAGCGAGAGATTATGGA	GCCTGATGCCAGGTTAAAGCA
*MIG1*	CACAAGGTGATAAAGCCAAGCA	GGTCGCTCTATAACAATGGCAC
*β-actin*	GTGCTATGTTGCTCTAGACTTCG	ATGCCACAGGATTCCATACC

**Table 2:** Primer sequences for RT-PCR.

Gene	Forward Primer (5′–3′)	Reverse Primer (5′–3′)
*Mig1*	TTGTTGGAGTTTTTGTTGGATTTA	AACTTCCTCATAATTCCCAACTTTAT
*β-actin*	GGCTGTATTCCCCTCCATCG	CCAGTTGGTAACAATGCCATGT

## Data Availability

No data were used for the research described in the article.
